# 2142. The use of *Pseudomonas aeruginosa* Phage PASA16 for non resolving infections

**DOI:** 10.1093/ofid/ofad500.1765

**Published:** 2023-11-27

**Authors:** Hadil Onallah, Ronen Hazan, Ran Nir-Paz

**Affiliations:** Hadassah-Hebrew University Medical Center, Jerusalem, Yerushalayim, Israel; The Hebrew University, Jerusalem, Yerushalayim, Israel; Hadassah-Hebrew University Medical Center, Jerusalem, Yerushalayim, Israel

## Abstract

**Background:**

A growing number of compassionate phage therapy use cases reported in the last decade, with only a limited number of clinical trials conducted and very few unsuccessful clinical trials reported. However, the limited understanding of individual clinical cases and lack of successful published clinical trials provides little evidence on the role of phages in refractory infections. Our objective was to bridge between single cases to early clinical trials and to describe the compassionate use of a single same phage - PASA16 - in sixteen patients with non-resolving *Pseudomonas aeruginosa* infections.

**Methods:**

We have collected all cases in which PASA16 was used and summarized clinical microbiology data, administration protocol, clinical data and outcome. In all instances involving intravenous phage administration (14 out of 16 cases), PASA16 phage was manufactured and provided *pro-bono* by Adaptive Phage Therapeutics.

**Results:**

PASA16 was administered either IV, locally to infection site or by topical use to 16 patients, with data available for 15 patients, mainly for osteoarticular and foreign device associated infections. Few minor side effects were noted including elevated liver function enzymes and a transient reduction in white blood cell count. Good clinical outcome was documented in 13 out of 15 (86%). Two clinical failures were reported.

**Conclusion:**

PASA16 with antibiotics was found to be relatively successful in patients who have failed previously in traditional approaches. Minimal successful duration of phage therapy was 8 days with once to twice a day administration. Such pre-phase 1 cohorts can outline the potential clinical protocols to be used and facilitate the design of future clinical trials.

**Disclosures:**

**Ran Nir-Paz, MD**, Adaptive phage theraputics: Supplied the phage product for this study|BiomX: Advisor/Consultant|Technophage: Grant/Research Support

